# VOICES FROM THE INSIDE: MEDICAL TREATMENT FURLOUGH IN THE LARGEST STATE PENITENTIARY IN US: LESSONS LEARNED

**DOI:** 10.1093/geroni/igad104.1606

**Published:** 2023-12-21

**Authors:** Jolie Harris

**Affiliations:** Louisiana State University Health Science Center New Orleans, New Orleans, Louisiana, United States

## Abstract

As part of the criminal justice reform to reduce prison population, the State of Louisiana undertook an initiative to explore a medical treatment furlough project. The plan was to transition long-term inmates from the prison to long-term care (LTC)). Angola, the largest maximum security prison in the U.S, had over 1000 prisoners 60 years or older. Eighty percent of the prison population is serving a life sentence. Prisoners with significant health and mobility issues were to be considered to be released to off-site facilities with parole supervision. We present lessons learned from exploring all aspects of a possible partnership between a Louisiana long-term care company and the correction department including: conducting site visits at Angola and the long-term care facility; gathering best practice data; exploring a referral process and acuity classification tool; exploring the role of the parole officer; and examining the prison population against the regulations for long-term care facilities from the perspective of the prisoners and current residents. Additional considerations were the impact on the local community and on-going support for services provided, along with staffing, staff training, and security and safety of the facility. After multiple discussions with representative from the Louisiana Department of Health we found the obstacles were too great to implement the program. Exposure to the challenges facing the corrections department and examining how to implement the Act as written was a lesson in the value of viewing an issue from multiple perspectives.

